# Emotional and visual responses to trypophobic images with object, animal, or human body backgrounds: an eye-tracking study

**DOI:** 10.3389/fpsyg.2024.1467608

**Published:** 2024-12-16

**Authors:** Pengfei Yu, Li Yu, Yuting Li, Cheng Qian, Jia Hu, Weiyi Zhu, Fang Liu, Qi Wang

**Affiliations:** ^1^School of Mental Health, Wenzhou Medical University, Wenzhou, Zhejiang, China; ^2^Xinchang Center for Disease Control and Prevention, Xinchang, Zhejiang, China; ^3^Institute of Mental Health and Drug Discovery, Oujiang Laboratory (Zhejiang Lab for Regenerative Medicine, Vision and Brain Health), Wenzhou, Zhejiang, China; ^4^Wenzhou Key Laboratory of Basic and Translational Research for Mental Disorders, Wenzhou Medical University, Wenzhou, Zhejiang, China; ^5^Affiliated Kangning Hospital, Wenzhou Medical University, Wenzhou, Zhejiang, China

**Keywords:** trypophobia, eye tracking, disgust, arousal, pupil

## Abstract

**Background:**

Trypophobia refers to the visual discomfort (e.g., disgust or anxiety) experienced by some people when viewing clusters of bumps or holes. The spectral profile framework suggests that the spectral components of clustered patterns induces trypophobia. In contrast, the cognitive framework speculates that cognitive appraisal of dangerous objects (e.g., ectoparasites) causes trypophobia. A background effect (e.g., more disgust toward trypophobic patterns on the skin than on a desk) seems to support the cognitive framework. However, there is no study providing objective evidence for that effect and verifying these frameworks at the same time. This study aims to address that limitation by psychometric and eye-tracking experiments.

**Methods:**

We recruited 183 participants from colleges. Initially, participants finished a personality questionnaire. The cohort then completed an eye-tracking experiment which showed the trypophobic pattern of lotus seed on three categories of background images (objects, animals and human bodies). Finally, participants rated the image’s disgust and arousal levels using a self-assessment rating scale. Meanwhile, we compared all images’ luminosity and power spectra.

**Results:**

Trypophobic images with the human body or animal backgrounds induced a higher level of disgust and arousal than those with the object backgrounds. Participants gazed faster and dwelled longer at the trypophobic patterns on human body images than on object or animal images. Furthermore, trypophobic images with human body or animal backgrounds induced more substantial pupil dilation than those upon object backgrounds. No significant difference was detected between the power of trypophobic images with human body backgrounds and objects backgrounds. As the trypophobic images with human body backgrounds induced significant emotional or visual responses compared to those with inanimate object backgrounds. Such inconsistent results imply that the differential emotional or visual responses to trypophobic images are probably not induced by the difference in power spectra. Finally, the disgust/arousal level toward trypophobic images did not correlate with personality traits.

**Conclusion:**

These results supported the background effect of trypophobia, namely, trypophobic images with animal or human body backgrounds induce more severe disgust and cause more arousal than those with object backgrounds. Our results support the cognitive, but not spectral profile, framework of trypophobia.

## 1 Introduction

Trypophobia refers to the visual discomfort (e.g., disgust or anxiety) experienced by some people when viewing clusters of bumps or holes, such as lotus seed heads or sponges (Imaizumi and Tanno, 2018; DiMattina et al., 2024). Previous studies have estimated that approximately 10–18% of adults have trypophobia tendencies, feeling disgusted toward trypophobic patterns (Cole and Wilkins, 2013; Wong et al., 2023; Cole et al., 2024). Trypophobia meets the criteria of the Diagnostic and Statistical Manual of Mental Disorders (DSM-5) for a specific phobia (American Psychiatric Association, 2013; Cole, 2024), with a phobia defined as ‘a marked, persistent, and excessive or unreasonable fear when the individual is exposed to a specific object or situation’. Individuals who have trypophobia also exhibit other common symptoms, such as disgust, fear, itchiness, goosebumps and nausea (Vlok-Barnard and Stein, 2017).

Generally, there are two frameworks posited to explain or understand trypophobia. One is the spectral profile framework, which supposes that trypophobia is induced by the specific spectral components of trypophobic images (Cole and Wilkins, 2013; Le et al., 2015; Sasaki et al., 2017). It has been reported that trypophobic images usually exhibit relatively high-contrast energy at mid-range spatial frequencies compared to non-trypophobic images, and the spectral characteristic of trypophobic patterns is similar to that of dangerous animals (for example, snakes or spiders, Cole and Wilkins, 2013; Sasaki et al., 2017). Based on these findings, some researchers emphasize the significance of spectral components’ involvement in causing trypophobia. However, the results of Pipitone and DiMattina (2020) show that the phase spectrum of trypophobic images, which determines the pattern of small clusters of objects, plays a much larger role than the amplitude spectrum in determining visual discomfort (Pipitone and DiMattina, 2020). This finding indicates that amplitude or power spectra is not the primary driver for trypophobic discomfort.

The second model for understanding trypophobia is the cognitive framework, which speculates that trypophobia is caused by cognitive appraisal of potentially dangerous objects that resemble ectoparasites or dermatoses (Yamada and Sasaki, 2017; Kupfer and Le, 2018; DiMattina et al., 2024). This framework is supported by the finding that participants with a history of skin problems rated the trypophobic pictures as evoking high discomfort compared to those without skin problems (Yamada and Sasaki, 2017). The authors of that study proposed the hypothesis of involuntary protection against dermatosis (IPAD), arguing that people probably associated trypophobic patterns with skin diseases, which further caused them to generate negative emotions. Thus, trypophobic stimuli would trigger an avoidance reaction toward potential pathogens (Yamada and Sasaki, 2017). Similarly, a previous study reported that priming with skin-problem-related words increased discomfort with trypophobic images (Shirai and Ogawa, 2021). The cognitive framework is also often discussed from an evolutionary perspective, emphasizing the instinctive protective response to skin-related diseases as an explanation of trypophobia (Yamada and Sasaki, 2017; Kupfer and Le, 2018). In brief, these studies highlighted the significance of cognitive appraisal in inducing trypophobia.

Further, the cognitive framework is also supported by some studies that reported intense disgust induced by trypophobic patterns on human skin backgrounds compared to other categories of backgrounds (Furuno et al., 2018; Pipitone et al., 2022). Thus, it seems that trypophobia has a background effect, namely, the degree of response to trypophobic images depends on the backgrounds of trypophobic patterns. As ectoparasites or dermatoses commonly exist in biotic organisms, a person might feel especially disgusted by an analog of ectoparasites or dermatoses in biotic organisms (Pipitone et al., 2022). However, there are some limitations of the former studies on the background effect in trypophobia: (1) the background effect was not obvious in the trypophobic images with non-dangerous animals background (Pipitone et al., 2022), which seems inconsistent with the evolutionary perspective of the cognitive framework of trypophobia. Considering that human beings can be infected with ectoparasites or dermatoses from animals, people should also show negative emotional responses to the trypophobic patterns on the skin of animals. This limitation of that former study may be caused by not using appropriate images (e.g., mammal images); (2) these studies only performed psychometric tests, and their results were somewhat subjective; (3) these studies did not rule out the effect of spectral components of trypophobic images. So, further experiments are needed to address these limitations and elucidate the background effect of trypophobia.

Eye tracking is a sensor technology that tracks eye movements and assesses what individuals are looking at in real-time by measuring the parameters of gaze/pupil and eye-movement trajectories. The gaze parameters primarily reflect the attention of individuals. Usually, individuals subconsciously allocate more time to gaze toward the visual stimuli that attract their attention. For instance, it has been found that infants allocate increased dwell time on fearful faces compared to happy faces (Segal and Moulson, 2020). In addition, a paper reported that trypophobic images attracted the individual’s attention to their location (Shirai et al., 2019). This study explores whether people pay more attention to trypophobic patterns on animal or human body backgrounds than object backgrounds.

Further, pupil size is an indicator of emotion, arousal, attention, and cognition (Viglione et al., 2023), which could be measured by an eye-tracking apparatus. The autonomic nervous system automatically adjusts pupil dilation or constriction according to exogenous or endogenous factors (Joshi and Gold, 2020). The sympathetic activation primarily drives pupil dilation, and the parasympathetic activation primarily drives pupil constriction (McDougal and Gamlin, 2015). For example, people’s pupils dilate when they see or know something scary or exciting (Prunty et al., 2022). However, it has been reported that trypophobic images elicited increased pupil constriction compared to neutral images (Ayzenberg et al., 2018). This study aims to test the background effect of trypophobia using pupillometry, assuming that pupil size dilates much more when trypophobic images with animal or human body backgrounds arouse people than with object backgrounds.

Personality reflects the tendency and sustainability characteristics that determine individual peculiarities in psychological behavior (such as thoughts, emotions and actions). Personality can be subdivided into many traits, such as the big five personality factors, including extraversion, agreeableness, conscientiousness, neuroticism and imagination (Donnellan et al., 2006). Personality traits are important factors that affect individual responses to stressful stimuli. For instance, persons with high levels of neuroticism tend to exhibit emotional instability (for example, becoming anxious or fearful) when facing a threat (Soliemanifar et al., 2018). Few studies reported on the relationship between personality traits and trypophobic responses. A previous study suggested that people with low levels of agreeableness felt more severe disgust of dense triangle patterns than those with high agreeableness levels (Zhang et al., 2021). A second paper showed no significant correlation between neuroticism and trypophobia (Kupfer and Le, 2018). This study also aims to investigate the correlation between personality traits and trypophobia.

Overall, this study aims to test the background effect of trypophobia using psychometric tests and eye-tracking experiment, which is expected to provide more objective evidence for the cognitive framework. Meanwhile, we compare the low-level visual properties (luminosity and power spectrum) of all images, which should also assist in validating the spectral profile framework. The hypothesis is that people would feel more severe disgust/arousal and allocate much more attention to trypophobic images with animal or human body backgrounds than with object backgrounds.

## 2 Materials and methods

### 2.1 Participants and procedure

Volunteer students aged 17–25 years (mean = 19.42, standard deviation = 1.55) were recruited from Wenzhou Medical University. The exclusion criteria for participation were: (1) have suffered from parasitic, dermal, ophthalmic, cardiac or mental diseases; (2) have studied parasitology, dermatology or anatomy; (3) did not complete the questionnaires or submitted questionnaires with logical errors (e.g., select the same rating for all items in a questionnaire which contains reverse items). Overall, 183 volunteers participated in our experiments.

**Figure 1:**
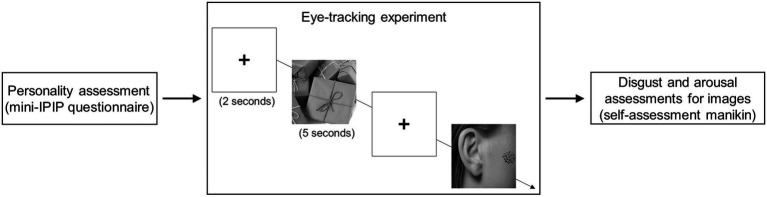
The flowchart of this study. Initially, participants finished a personality questionnaire and completed the eye-tracking experiment. Finally, they rated their disgust and arousal levels for each image presented in the eye-tracking experiment. Image sources: https://xsj.699pic.com/tupian/1opz28.html, https://xsj.699pic.com/tupian/1yv1w9.html. Reproduced with permission.

The experimental procedure were: Initially, participants completed a personality questionnaire (mini-IPIP). Then, they participated in the eye-tracking experiment. Finally, they were asked to rate their disgust and arousal levels for each image presented in the eye-tracking experiment (Figure 1).

An eye-tracking apparatus (Tobii TX300, Sweden) emits infrared light to the eyes of a subject and receives the reflected infrared light of the eyes, by which the apparatus computes the positon and movement of the eyes. Due to participants blinking or moving their heads during recording, it is unavoidable to lose a certain extent of signal. At the end of recording, the apparatus can show the sampling rate (i.e., the proportion of the received reflection signal to the total transmitted signal). Higher sampling rate means higher level of data fidelity. To guarantee the quality of our analysis, we set the criterion of data collection at sampling rate ≥ 90%. Overall, 183 participants completed all experiments. Of the cohort, 76 volunteers reached the high-quality criterion of the eye-tracking experiment (sampling rate ≥ 90%).

### 2.2 The mini international personality item pool (mini-IPIP)

The mini-IPIP is a questionnaire consisting of 20 items that measure the five factors of personality (Donnellan et al., 2006): extraversion (e.g., “I am the life of the party”), agreeableness (e.g., “I feel others’ emotions”), conscientiousness (e.g., “I like order”), neuroticism (e.g., “I get upset easily”) and imagination/intellect (e.g., “I have a vivid imagination”). These five factors are each measured by four items with a five-point Likert scale ranging from 1 (strongly disagree) to 5 (strongly agree). The mini-IPIP has good validity and reliability (Li et al., 2012; Cronbach’s *α* coefficient for each factor in the current study: extraversion = 0.732, agreeableness = 0.755, conscientiousness = 0.648, neuroticism = 0.805, imagination/intellect = 0.674).

### 2.3 Visual stimuli

**Figure 2:**
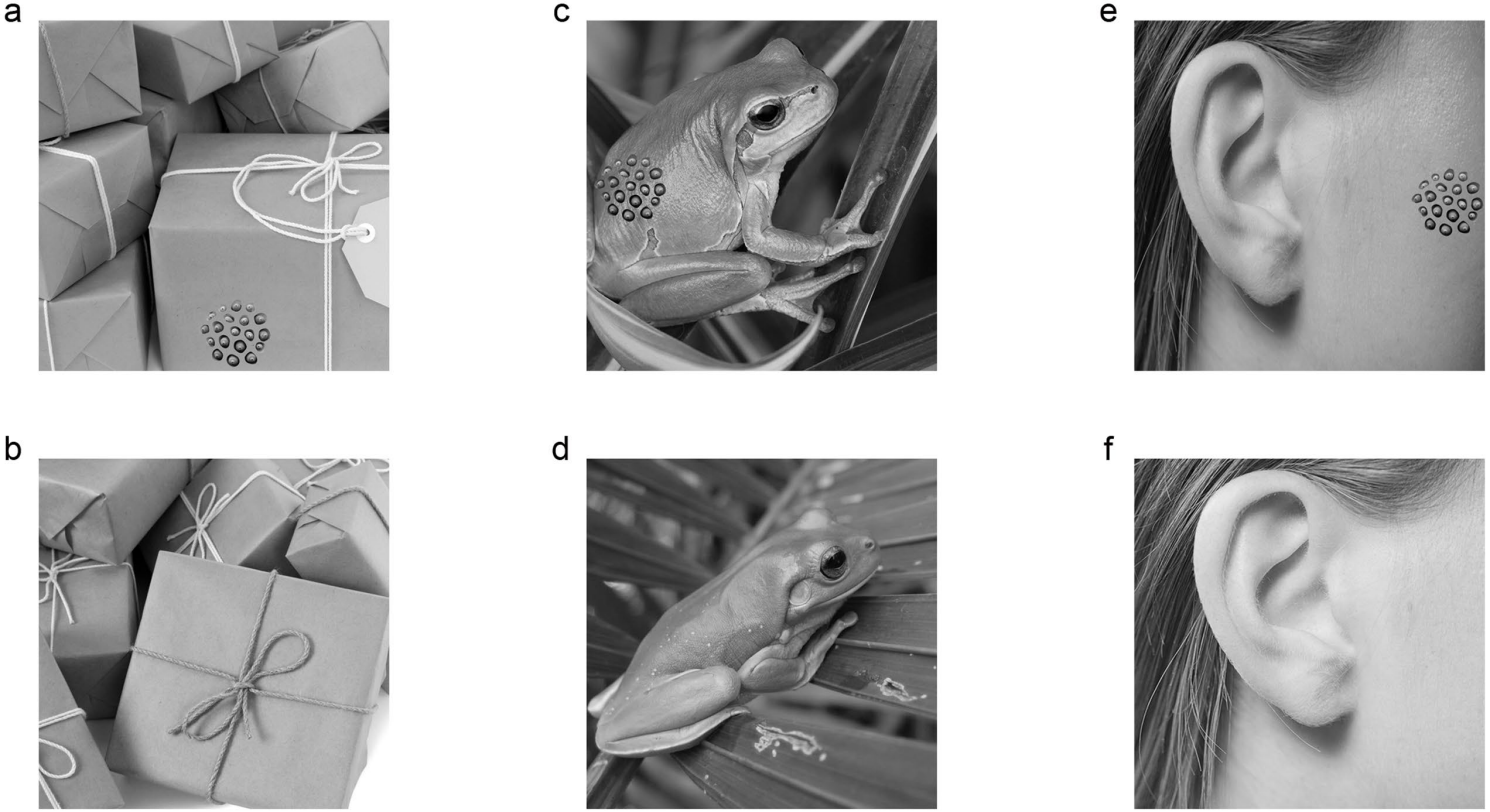
Representative trypophobic images and their matched control images used in this study are shown. (a) Trypophobic image with object background. (b) Control image with object background. (c) Trypophobic image with animal background. (d) Control image with animal background. (e) Trypophobic image with human body background. (f) Control image with human body background. Images sources: https://xsj.699pic.com/tupian/1opz29.html, https://xsj.699pic.com/tupian/1opz28.html, https://xsj.699pic.com/tupian/0vuv96.html, https://xsj.699pic.com/tupian/0h09ln.html, https://xsj.699pic.com/tupian/1yv1w9.html, https://xsj.699pic.com/tupian/22vu1k.html. Reproduced with permission.

Initially, we tailored a pattern of lotus seed from an image of lotus seed head by the “lasso tool” of Photoshop software (Adobe Photoshop, United States). Then, we created trypophobic images by pasting the pattern of lotus seed (7.3 cm × 7.3 cm) on three categories of background images (Figure 2, size: 36 cm × 36 cm): (1) the object group included a mango, tomato, bell pepper, egg, sofa, pillow, gift box and plank; (2) the animal group included a camel, horse, gazelle, dog, dolphin, penguin, fish and frog; (3) the human body images included a face, tongue, arm, abdomen, leg and foot. To eliminate the bias of gaze direction, the trypophobic pattern of lotus seed was separately pasted on four different locations (i.e., right, left, top or bottom) of background images. Each control image was similar to its matched trypophobic image but did not contain lotus seed pattern (Figure 2). Finally, we converted the images to luminance (shortcut: ctrl+shift+u) and transformed to a resolution of 786 × 768 pixels using the Photoshop software (Adobe Photoshop, United States).

**Figure 3:**
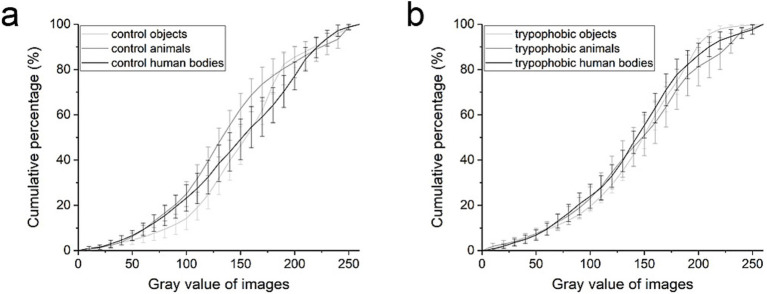
Cumulative curves of gray value for control images (a) and trypophobic images (b). Control or trypophobic objects (light gray), control or trypophobic animals (gray), and control or trypophobic human bodies (black).

To compare the luminance of different kinds of images, the rgb2gray function of MATLAB software (MathWorks, United States) was used to calculate the gray value of each image and, respectively, plotted the cumulative curves of gray values for trypophobic and control images (Figure 3). As shown in Figures 3a,b, respectively, there is no significant difference between the cumulative curves of gray value for control and trypophobic images [Figure 3, repeated measures ANOVA, *p* > 0.05 for each checking window (10 bin, 10 steps), *n* = 8 for each curve].

**Figure 4:**
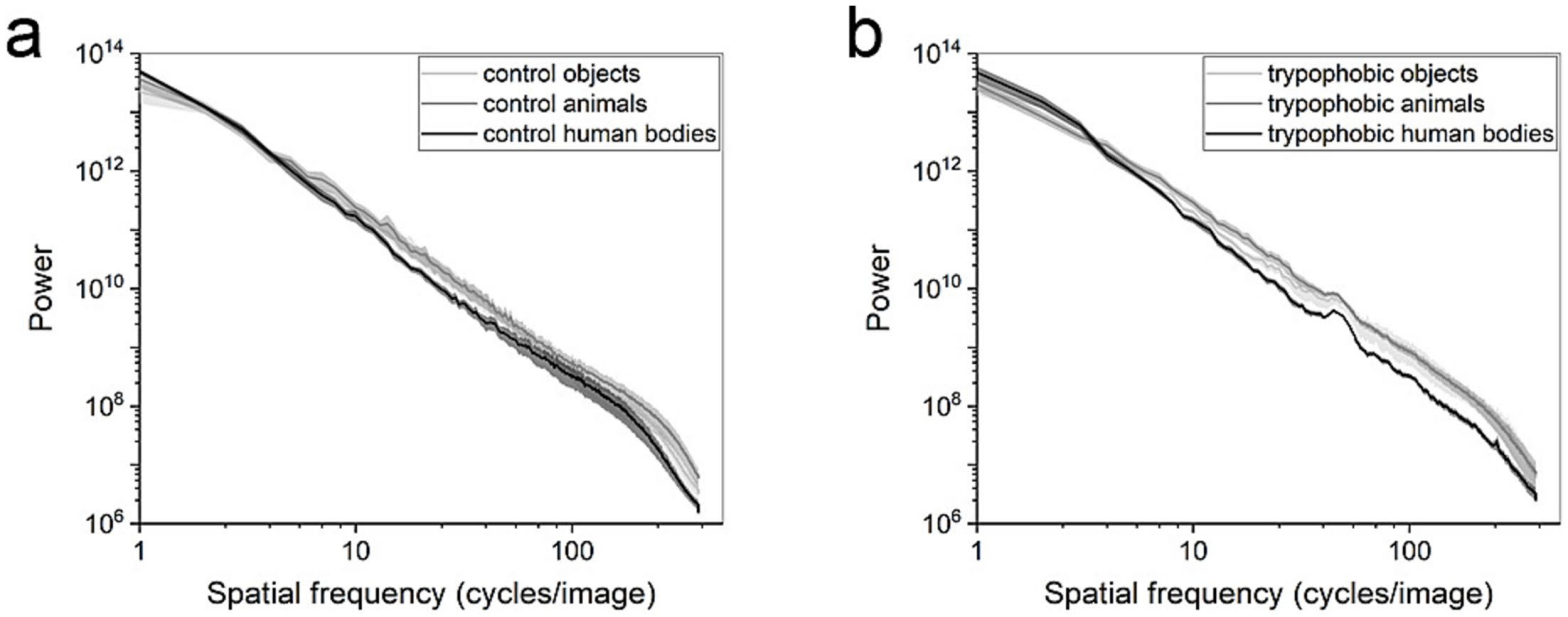
Log–log plots of power spectra for (a) control images and (b) trypophobic images. Control or trypophobic objects (light gray), control or trypophobic animals (gray), and control or trypophobic human bodies (black).

To compare the spectral components of different image types, the SHINE toolbox was used to calculate the power spectra for every kind of images (Figure 4, Willenbockel et al., 2010). As shown in Figure 4a, there is no significant difference in the power of control images in all the ranges of spatial frequency (Figure 4a, repeated measures ANOVA, *p* > 0.05, *n* = 8). With respect to the trypophobic images, there is only a significant difference between the power of trypophobic images with human body and animal backgrounds in the (20–132 cycles per image) range of spatial frequency [Figure 4b, paired *t*-test, *p* < 0.05 for each checking window (one bin, one step), *n* = 8]. No significant difference is detected between the power of trypophobic images with human body backgrounds and object backgrounds at all the spatial frequency ranges assessed (Figure 4b, paired *t*-test, *p* > 0.05).

### 2.4 Eye-tracking apparatus and protocol

Gazing and pupil dilation data were recorded using an eye-tracking apparatus (Tobii TX300, Sweden) at a frequency of 300 Hz with an accuracy of 0.4–0.6°. The desk-mounted remote infrared eye tracker allows head movements within 34 cm × 26 cm at 65 cm (horizontal × vertical × depth) without accuracy reduction. All visual stimuli were presented on a 23-inch monitor. Prior to a test, an in-built five-point calibration procedure was performed. The distance between the participant and the apparatus was approximately 65 cm.

When a test initiates, a fixation cross (2 s) appears in the middle area of the monitor. Then, a trypophobic image or control image was randomly shown for 5 s in the middle area of the monitor (Figure 1). The eye-tracking protocol terminated until the sequential pattern had shown all images “a fixation cross + an image + a fixation cross + an image ….” The trypophobic pattern (i.e., the area of lotus seed) was defined as the area of interest (AOI) in the eye-tracking experiment. Approximatively, the visual angle of a displayed image is 31°, and the visual angle of the trypophobic pattern is 6.4°.

For the eye-tracking experiment, several parameters were used to quantify the gazing response to the area of interest (AOI) of trypophobic images: (1) ‘first fixation latency’ refers to the interval between the presentation of a trypophobic image and the first fixation attending at its trypophobic AOI; (2) ‘first fixation duration’ refers to the duration of the first gaze at a trypophobic AOI; (3) ‘fixation count’ refers to the number of all gazes at a trypophobic AOI; (4) ‘dwell time’ refers to the summed duration of fixations attending at a trypophobic AOI. In addition, the parameter ‘relative pupil dilation’ was used to measure the change in pupil size. ‘Relative pupil dilation’ was calculated by subtracting the pupil diameter of control images from that of matched trypophobic images.

### 2.5 Self-assessment of emotional responses

Based on the self-assessment manikin (SAM), a pictorial assessment technique that measures the pleasure and arousal associated with a person’s emotional reaction to stimuli (Bradley and Lang, 1994), a self-report rating scale was used to estimate participants’ disgust and arousal levels for each image. A nine-point rating scale ranging from 1 (extremely disgust or calm) to 9 (extremely pleasure or arousal) was implemented.

For the three groups of trypophobic images (i.e., with an object, animal or human body background), a “relative disgust score” was calculated by subtracting the disgust rating of trypophobic images from matched control images. A “relative arousal score” was calculated by subtracting the arousal rating of control images from matched trypophobic images.

### 2.6 Statistical analysis

Data was analyzed using SPSS software (IBM SPSS Statistics, United States) and R software (R Core Team).^1^ The Shapiro–Wilk test for normality was performed on the data (Supplementary material). For abnormally distributed data (Shapiro–Wilk test: *p* < 0.05), we used the Friedman or Wilcoxon tests to detect the significance of differences between the groups. For normally distributed data (Shapiro–Wilk test: *p* > 0.05), we used the repeated measures ANOVA or paired *t*-test to detect the significance of differences between the groups (Mishra et al., 2019); meanwhile, we also built a linear mixed-effects model to investigate the impact of image categories on pupillary response by the “lme4” package of R software. In the linear mixed-effects model, we treated the categories of trypophobic images as fixed effect, while participant identity (gender and age) as random effect. The significance level for these analyses was set at 0.05 (*p < 0.05, **p < 0.01, ***p < 0.001). *p*-values were Bonferroni corrected for multiple comparisons. Data in the text and figures were expressed as means ± SEM (standard error of the mean).

## 3 Results

### 3.1 Emotional rating on trypophobic images with different backgrounds

**Figure 5:**
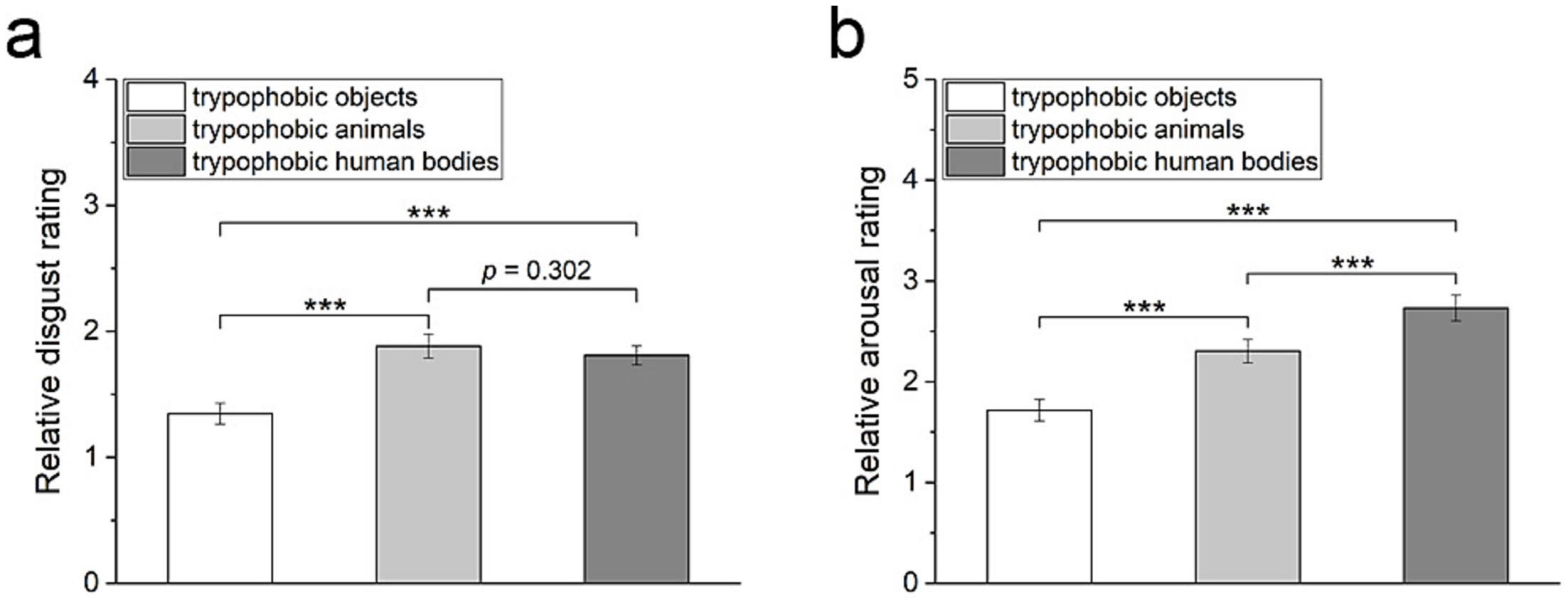
Comparison of the relative disgust rating (a) and the relative arousal rating (b) in response to trypophobic images with three categories of backgrounds. *p*-values were Bonferroni corrected for multiple comparisons. Wilcoxon test, ***p < 0.001.

The relative disgust/arousal levels felt toward trypophobic images with three categories of backgrounds are compared to estimate the impact of background images on emotional responses to trypophobic patterns. As shown in Figure 5a, there is a significant difference between the relative disgust scores among these three groups of trypophobic images (Friedman test, Kendall’s *W* = 0.14, *χ*^2^ = 49.92, *p* < 0.001, *n* = 183). Specifically, the relative disgust scores of trypophobic images with a human body or animal backgrounds are significantly larger than those with inanimate objects backgrounds, respectively (Figure 5a, Wilcoxon test, Cohen’s *d* = 0.43 and 0.45 respectively, *z* = −5.71 and −7.26 respectively, *p* < 0.001 and *n* = 183 for both).

Additionally, there is also a significant difference between the relative arousal scores among these three groups of trypophobic images (Figure 5b, Friedman test, Kendall’s *W* = 0.24, *χ*^2^ = 89.11, *p* < 0.001, *n* = 183). Pairwise comparisons between these three groups reveal that the trypophobic images with human body backgrounds have the most substantial relative arousal scores. In contrast, the trypophobic images with goods backgrounds have the lowest relative arousal scores (Figure 5b, Wilcoxon test, trypophobic human bodies vs. trypophobic animals: Cohen’s *d* = 0.26, *z* = −5.94, *p* < 0.001; trypophobic human bodies vs. trypophobic objects: Cohen’s *d* = 0.64, *z* = −8.70, *p* < 0.001; trypophobic animals vs. trypophobic objects: Cohen’s *d* = 0.39, *z* = −7.11, *p* < 0.001). These results confirm that the background of trypophobic pattern has an evident impact on emotional responses.

### 3.2 Gazing responses to trypophobic images with different backgrounds

**Figure 6:**
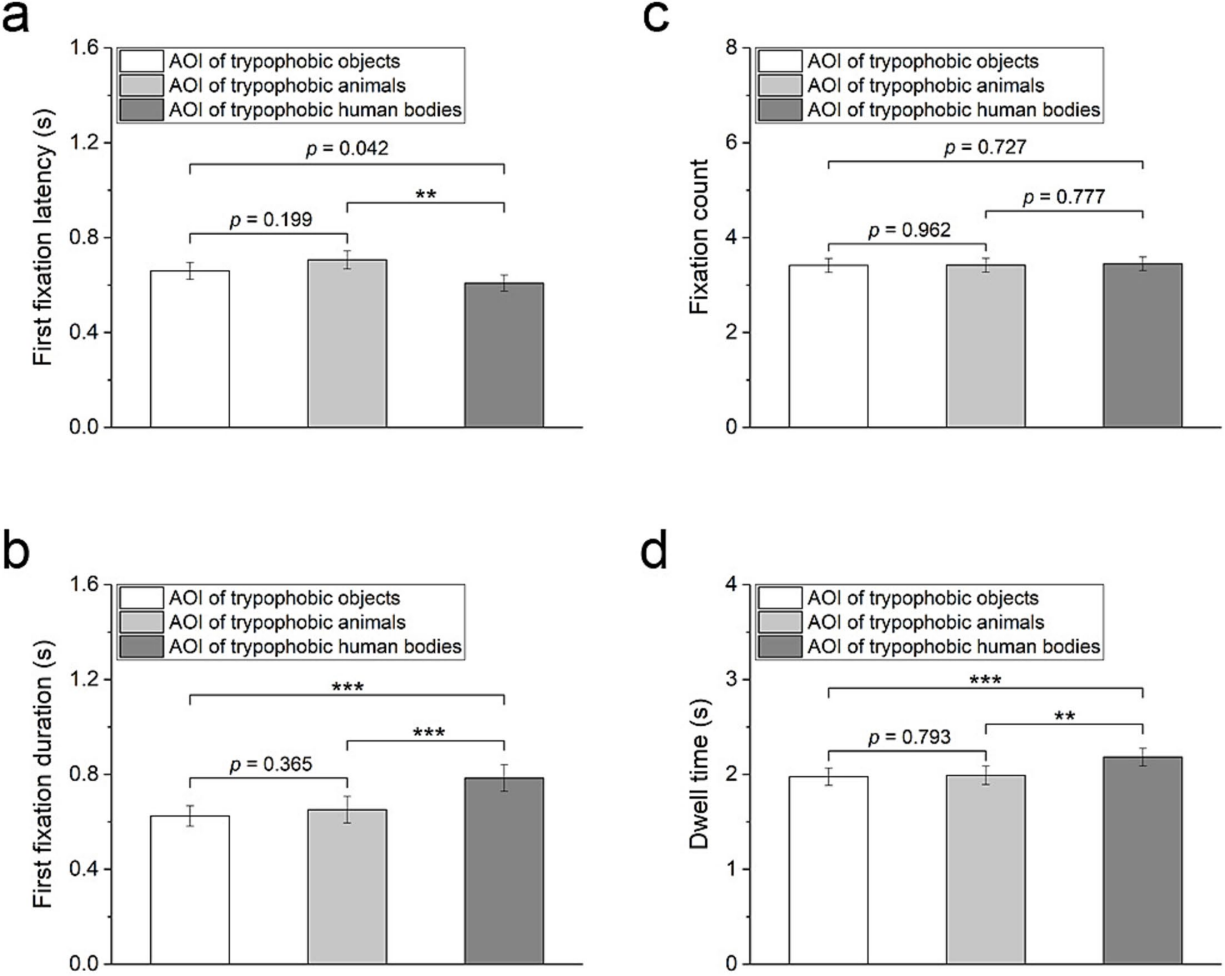
Comparison of gazing responses to trypophobic images with three categories of backgrounds. Briefly, trypophobic images with human body backgrounds have the smallest value of ‘first fixation latency’ (a), the largest value of ‘first fixation duration’ (b), and the largest value of ‘dwell time’ (d). There is no significant difference in the ‘fixation count’ values among the three trypophobic images (c). AOI, area of interest of trypophobic images (i.e., the area of lotus seed). Wilcoxon test for (a, b) and Paired *t*-test for (c, d), *p*-values were Bonferroni corrected for multiple comparisons. **p < 0.01, ***p < 0.001.

The gazing parameters toward trypophobic patterns on the three categories of background images are compared to estimate the impact of the background image on attention responses to trypophobic patterns. Significant differences in the “first fixation latency,” “first fixation duration,” and “dwell time” among these three groups are identified (Figure 6a, Friedman test, Kendall’s *W* = 0.11, *χ*^2^ = 17.13, *p* < 0.001; Figure 6b, Friedman test, Kendall’s *W* = 0.12, *χ*^2^ = 18.62, *p* < 0.001; Figure 6d, repeated measures ANOVA, *η*^2^ = 0.11, *F*_2,76_ = 8.76, *p* < 0.001). However, there is no significant difference in the “fixation count” among these three groups (Figure 6c, repeated measures ANOVA, *η*^2^ = 0.001, *F*_2,76_ = 0.07, *p* = 0.94).

Notably, the first fixation latency is significantly shorter for the trypophobic patterns (the AOI) on a human body image than on an animal image (Figure 6a, Wilcoxon test, Cohen’s *d* = 0.32, *z* = −3.42, *p* < 0.01) and also relatively shorter than on an object background image (Figure 6a, Wilcoxon test, Cohen’s *d* = 0.17, *z* = −2.04, *p* = 0.04). Additionally, compared to the trypophobic patterns on animal or object background images, the trypophobic patterns on human body background images have a longer first fixation duration (Figure 6b, Wilcoxon test, trypophobic human bodies vs. trypophobic animals: Cohen’s *d* = 0.27, *z* = −4.30, *p* < 0.001; trypophobic human bodies vs. trypophobic objects: Cohen’s *d* = 0.37, *z* = −4.00, *p* < 0.001) and longer dwell time (Figure 6d, paired *t*-test, trypophobic human bodies vs. trypophobic animals: Cohen’s *d* = 0.38, *t* = 3.35, *p* < 0.01; trypophobic human bodies vs. trypophobic objects: Cohen’s *d* = 0.47, *t* = 4.12, *p* < 0.001). These results indicate that the background of trypophobic patterns has a moderate impact on attention responses.

### 3.3 Pupillary responses to trypophobic images with different backgrounds

**Figure 7:**
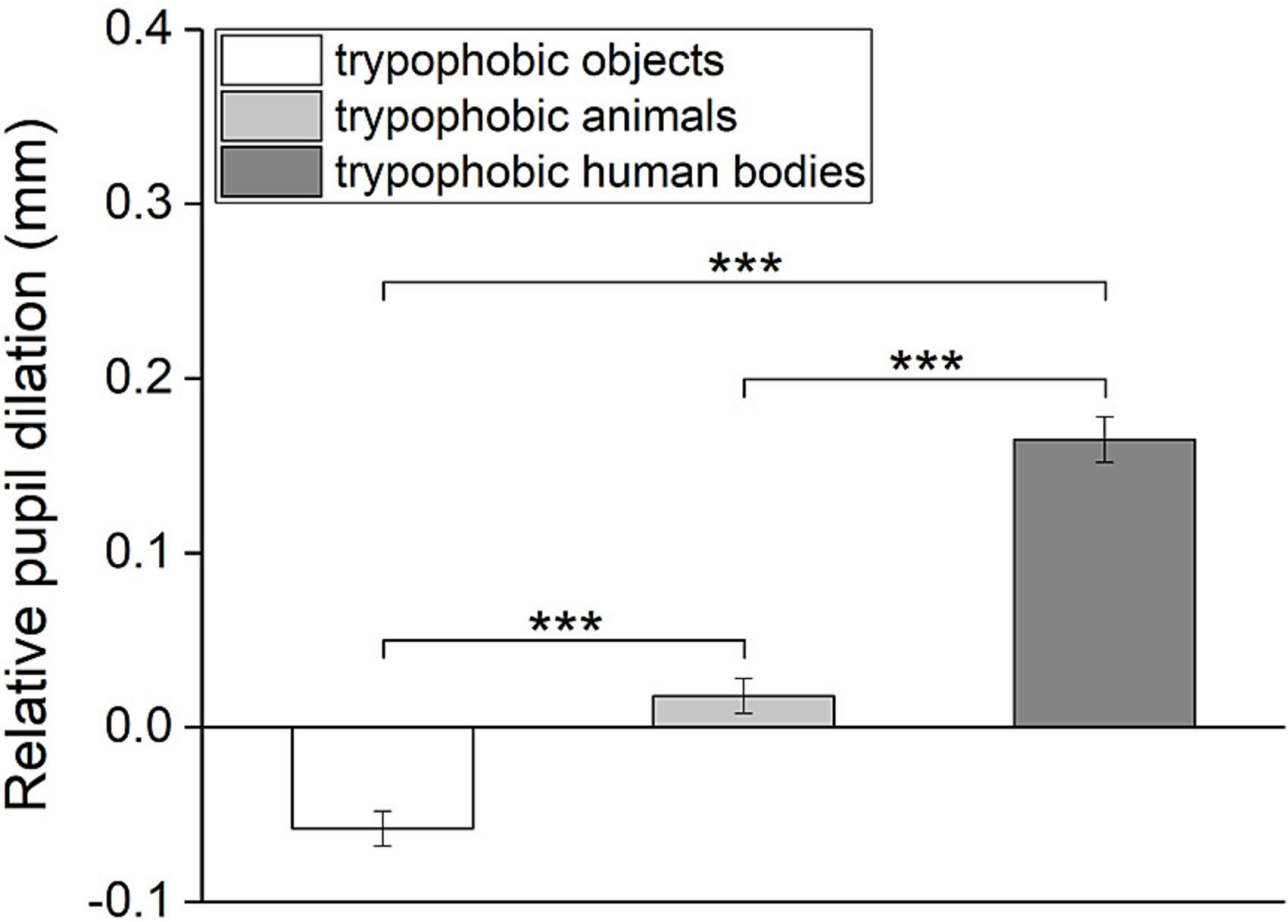
Comparison of the relative pupil dilation for trypophobic images with three categories of backgrounds. *p*-values were Bonferroni corrected for multiple comparisons. Paired *t*-test, ***p < 0.001.

Changes in pupil size toward trypophobic images with three categories of backgrounds are compared to test the impact of the background image on pupillary responses to trypophobic patterns. As shown in Figure 7, there is a significant difference between the relative pupil dilation among these three groups (Repeated measures ANOVA, *η*^2^ = 0.56, *F*_2,76_ = 94.99, *p* < 0.001). *Post hoc* paired *t*-test reveal that the relative pupil dilation to trypophobic images with human body backgrounds is significantly larger than with animal or object backgrounds (Figure 7, trypophobic human bodies vs. trypophobic animals: Cohen’s *d* = 1.19, *t* = −10.35, *p* < 0.001; trypophobic human bodies vs. trypophobic objects: Cohen’s *d* = 1.37, *t* = −11.98, *p* < 0.001). Meanwhile, the relative pupil dilation to trypophobic images with animal backgrounds is also significantly higher than that with the object backgrounds (Figure 7, Cohen’s *d* = 0.53, *t* = −4.66, *p* < 0.001).

Similarly, results from the linear mixed-effects model revealed significant main effect of image categories on relative pupil dilation (Table 1, estimate = 0.11, SE = 0.01, *p* < 0.001). Pairwise comparisons showed significant difference between these three categories (Table 1, trypophobic human bodies vs. trypophobic objects: estimate = 0.22, SE = 0.02, *p* < 0.001; trypophobic human bodies vs. trypophobic animals: estimate = 0.15, SE = 0.02, *p* < 0.001; trypophobic animals vs. trypophobic objects: estimate = 0.08, SE = 0.02, *p* < 0.001). Taken together, these results indicate that the background of trypophobic patterns greatly influences the change in pupil size.

**Table 1:** Linear mixed-effects model results evaluating effect of image categories on relative pupil dilation.

	Estimate	SE	95% CI	*p*
Intercept	−0.18	0.02	(−0.21, −0.15)	<0.001
Image categories	0.11	0.01	(0.10, 0.13)	<0.001
TRY human bodies vs. TRY objects	0.22	0.02	(0.19, 0.26)	<0.001
TRY human bodies vs. TRY animals	0.15	0.02	(0.11, 0.18)	<0.001
TRY animals vs. TRY objects	0.08	0.02	(0.04, 0.11)	<0.001

TRY human bodies, trypophobic human bodies; TRY animals, trypophobic animals; TRY objects, trypophobic objects; SE, standard error; CI, confidence interval.

### 3.4 Correlation between trypophobia and personality traits

The correlation between trypophobia and personality traits was explored, however, no significant correlation between the relative disgust/arousal score to trypophobic images and the five traits of personality (i.e., extraversion, agreeableness, conscientiousness, neuroticism and imagination), respectively (Table 2, Spearman correlation test, *p* > 0.01 for all, *n* = 183) is detected.

**Table 2:** Correlations between relative disgust/arousal score toward trypophobic images and personality traits.

		Extraversion	Agreeableness	Conscientiousness	Neuroticism	Imagination
Disgust	Correlation coefficient	0.06	0.08	0.02	−0.03	−0.03
	*P-*value	0.40	0.31	0.75	0.74	0.73
Arousal	Correlation coefficient	0.02	0.15	0.13	−0.08	−0.03
	*P-*value	0.78	0.05	0.08	0.31	0.73

Correlation coefficients and *p-*value are shown. Spearman correlation test. The significance level was set at 0.01 (Bonferroni corrected for multiple comparisons).

## 4 Discussion

The psychometric results demonstrate that the trypophobic image with an animal or human body background induced relatively higher levels of disgust and arousal in participants compared to that with an object background. The overall outcomes are primarily consistent with previous studies that report an intense disgust induced by trypophobic patterns on human skin backgrounds compared to other categories of backgrounds (Furuno et al., 2018; Pipitone et al., 2022). Especially, our findings address the first limitation mentioned in our introduction (fourth paragraph) and verify the background effect of trypophobia. Our findings also support the evolutionary perspective of the cognitive framework of trypophobia. Given that disgust is regarded as a main adaptation for defending against pathogens and parasites in humans (Kupfer and Fessler, 2018), it seems that the severe disgust felt toward the specific combination of a trypophobic pattern with biotic backgrounds facilitates the emotional response to infection danger (e.g., ectoparasites or dermatoses), lending support to the cognitive framework of trypophobia (Yamada and Sasaki, 2017; Kupfer and Le, 2018).

The eye-tracking results show that trypophobic patterns on human body background images attracted individuals’ attention most sensitively and intensely, as indicated by the shorter latency and longer dwell time in reaction to the trypophobic patterns, respectively. Although it has been demonstrated that trypophobic images induce a faster visual process than non-trypophobic control images (Shirai and Ogawa, 2019). Our present study specifically elucidate that background categories exerted a substantial impact on visual response to trypophobic patterns. Our findings address the second limitation mentioned in our introduction (fourth paragraph) and provide more objective evidence for the background effect of trypophobia. Considering that early awareness of danger can help individuals respond faster, the specific sensitive gazing responses to trypophobic patterns on human body background images imply that this type of trypophobic image facilitates attention to infectious danger and contains survival value.

The data demonstrate that relative to matched control images, trypophobic images with animal or human body backgrounds induce a low or high level of pupil dilation, respectively. In contrast, the trypophobic images with object backgrounds induced pupil constriction. Our findings are partially consistent with a previous study, when researchers merely used trypophobic images, specifically trypophobic patterns with no apparent backgrounds, to elicit pupil constriction compared to neutral images (Ayzenberg et al., 2018). It has been declared that fear stimuli induced pupil dilation and disgust stimuli induced pupil constriction (Wang et al., 2021). But many studies demonstrated that individuals showed larger pupil dilation during viewing emotionally arousal stimuli regardless of the valence (reflecting the positive or negative value) of stimuli, indicating that pupillary response reflects emotional arousal, but not emotional valence (Bradley et al., 2008, 2017; Ferencova et al., 2021; Henderson et al., 2018). Our findings were consistent with that viewpoint, as the most arousing images (trypophobic human bodies) induced the highest level of pupil dilation in our study.

We did not detect a significant difference between the power of trypophobic images with human body backgrounds and objects backgrounds, nor between the power of trypophobic images with animal backgrounds and objects backgrounds. As the trypophobic images with human body or animal backgrounds induced significant emotional or visual responses compared to those with object backgrounds. Such inconsistent results imply that the differential emotional or visual responses to trypophobic images are probably not induced by the difference in power spectra. Our findings address the third limitation mentioned in our introduction (fourth paragraph), ruling out the inducement of power spectra for the background effect of trypophobia. Two previous studies also reported similar results. For example, it has been shown that the trypophobic images still induce discomfort, even when the excess energy at midrange spatial frequencies is removed (Le et al., 2015; Pipitone and DiMattina, 2020). A recent study found that low-level image statistics were poor predictors of the visual comfort elicited by natural textures, including trypophobic and disease imagery (DiMattina et al., 2024). In combination, these overall results show that the background effect of trypophobia is not due to the difference in the power spectra of images. These overall results do not support the spectral profile framework of trypophobia.

In the present study, we controlled the luminance and category of images to guarantee our conclusions. Meanwhile, considering that some control images (such as dog or tongue) also lead to noticeable emotional or pupillary fluctuations, the emotional and pupillary responses toward trypophobic images were normalized by subtracting the corresponding responses toward matched control images. However, the present study participants were not ignorant about parasites and dermatoses, possibly suggesting that the emotional and visual responses toward trypophobic images are based on acquired knowledge but not instinctive property. Therefore, it is necessary to perform similar tests in parasite- and dermatosis-naive individuals (e.g., infants or children) to further clarify the instinct hypothesis of trypophobia (Suzuki et al., 2023).

Finally, no significant correlation was detected between the big five traits of personality and emotional responses to trypophobic images. However, it has been argued that personality traits affect an individual’s responses to stressful stimuli (Soliemanifar et al., 2018). The data from the current study are consistent with those from a previous study, which only tested the correlation between neuroticism and trypophobia, finding no significance (Kupfer and Le, 2018). The current findings indicate that trypophobia is a stable intrinsic peculiarity that is not influenced by personality traits.

## 5 Conclusion

This study primarily demonstrate that the trypophobic images with animal or human body backgrounds induce more intense disgust, cause more arousal, and attract more attention than those with object backgrounds. Here, we provide more objective evidence for the background effect of trypophobia and the evolutionary perspective of the cognitive framework of trypophobia. Meanwhile, the spectral comparison data indicate that the background effect of trypophobia is not due to the difference in the power spectra of images. Overall, our findings support the cognitive, but not spectral profile, framework of trypophobia. These results imply that the visual and emotional responses to the specific combination of a trypophobic pattern with an organism, especially human body, background plays an essential role in facilitating the attentional awareness and emotional defense to infection danger (e.g., ectoparasites or dermatoses) and, thus, contain survival value.

## Data Availability

The raw data supporting the conclusions of this article will be made available by the authors, without undue reservation.

## Footnotes

^1^ The R Project for statistical computing. https://www.r-project.org/
