# Supplementary material for: Emotional and visual responses to trypophobic images with object, animal, or human body backgrounds: an eye-tracking study
Source: Front Psychol. 2024 Dec 16;15:1467608. doi: 10.3389/fpsyg.2024.1467608 (PMC11684096; doi:10.3389/fpsyg.2024.1467608)
Supplement: Supplementary file 1 [file Data_Sheet_1.doc]

**Supplementary material**

Shapiro-Wilk test for three categories of Figure 5

|  |  | Relative disgust rating | | |  | Relative arousal rating | | |
| --- | --- | --- | --- | --- | --- | --- | --- | --- |
|  |  | *W* |  | *p* |  | *W* |  | *p* |
| Trypophobic objects |  | 0.938 |  | < 0.001 |  | 0.94 |  | < 0.001 |
| Trypophobic animals |  | 0.969 |  | < 0.001 |  | 0.979 |  | < 0.01 |
| Trypophobic human bodies |  | 0.979 |  | < 0.01 |  | 0.978 |  | < 0.01 |

Shapiro-Wilk test for three categories of Figure 6

|  |  | First fixation latency | | |  | First fixation duration | | |  | Fixation count | | |  | Dwell time | | |
| --- | --- | --- | --- | --- | --- | --- | --- | --- | --- | --- | --- | --- | --- | --- | --- | --- |
|  |  | *W* |  | *p* |  | *W* |  | *p* |  | *W* |  | *p* |  | *W* |  | *p* |
| AOI of trypophobic objects |  | 0.848 |  | < 0.001 |  | 0.693 |  | < 0.001 |  | 0.98 |  | 0.267 |  | 0.974 |  | 0.128 |
| AOI of trypophobic animals |  | 0.885 |  | < 0.001 |  | 0.67 |  | < 0.001 |  | 0.974 |  | 0.118 |  | 0.957 |  | 0.012 |
| AOI of trypophobic human bodies |  | 0.854 |  | < 0.001 |  | 0.751 |  | < 0.01 |  | 0.968 |  | 0.054 |  | 0.977 |  | 0.18 |

AOI: area of interest.

Shapiro-Wilk test for three categories of Figure 7

|  |  | Relative pupil dilation | | |
| --- | --- | --- | --- | --- |
|  |  | *W* |  | *p* |
| Trypophobic objects |  | 0.992 |  | 0.905 |
| Trypophobic animals |  | 0.963 |  | 0.027 |
| Trypophobic human bodies |  | 0.976 |  | 0.153 |

Shapiro-Wilk test for five personality traits of Table 1

|  |  | extraversion |  | agreeableness |  | conscientiousness |  | neuroticism |  | imagination |
| --- | --- | --- | --- | --- | --- | --- | --- | --- | --- | --- |
| W |  | 0.974 |  | 0.951 |  | 0.964 |  | 0.982 |  | 0.973 |
| *p* |  | < 0.01 |  | < 0.001 |  | < 0.001 |  | < 0.05 |  | < 0.01 |
